# Analysis of current and alternative phenol based RNA extraction methodologies for cyanobacteria

**DOI:** 10.1186/1471-2199-10-79

**Published:** 2009-08-07

**Authors:** Fernando Lopes Pinto, Anders Thapper, Wolfgang Sontheim, Peter Lindblad

**Affiliations:** 1 Department of Photochemistry and Molecular Science, The Ångström Laboratories, Uppsala University, Box 523, SE-75120, Uppsala, Sweden

## Abstract

**Background:**

The validity and reproducibility of gene expression studies depend on the quality of extracted RNA and the degree of genomic DNA contamination. Cyanobacteria are gram-negative prokaryotes that synthesize chlorophyll *a *and carry out photosynthetic water oxidation. These organisms possess an extended array of secondary metabolites that impair cell lysis, presenting particular challenges when it comes to nucleic acid isolation. Therefore, we used the NHM5 strain of *Nostoc punctiforme *ATCC 29133 to compare and improve existing phenol based chemistry and procedures for RNA extraction.

**Results:**

With this work we identify and explore strategies for improved and lower cost high quality RNA isolation from cyanobacteria. All the methods studied are suitable for RNA isolation and its use for downstream applications. We analyse different Trizol based protocols, introduce procedural changes and describe an alternative RNA extraction solution.

**Conclusion:**

It was possible to improve purity of isolated RNA by modifying protocol procedures. Further improvements, both in RNA purity and experimental cost, were achieved by using a new extraction solution, PGTX.

## Background

Cyanobacteria are gram-negative prokaryotes that synthesize chlorophyll *a *and carry out photosynthetic water oxidation [1]. Since they have simple nutritional requirements, needing only air, water and mineral salts, with light as the only energy source [2], their potential industrial application is significant – from e.g. hydrogen production [3,4] to various biotechnological purposes [5].

In order to develop this biotechnological potential, it is important to thoroughly understand different aspects of cyanobacterial physiology and metabolism. As a part of such a process, obtaining reliable gene expression data is vital. Several methods, from Northern blotting to microarrays, are routinely used to obtain such data. The validity and reproducibility of the data obtained depend on the quality of the extracted RNA and the degree of genomic DNA contamination. However, cyanobacteria present particular challenges when it comes to nucleic acid isolation – these organisms possess an extended array of secondary metabolites [6] that impair e.g. cell lysis and nucleic acid purification [7].

In order to assess the quality of RNA preparations two strategies are commonly followed: spectrophotometric analysis and ribosomal integrity verification by electrophoresis.

From the spectrophotometric analysis three absorbance values usually are taken into consideration – 230, 260 and 280 nm. The ratio between the absorbance at 260 nm and 280 nm is used to evaluate the purity of the nucleic acid – for "pure" RNA a ratio around 2.0 is expected. A lower ratio may indicate the presence of proteins and peptides absorbing around 280 nm. Additionally, the ratio between the absorbance at 260 nm and 230 nm is expected to be 2.2 for "pure" nucleic acid samples. A lower ratio might be the consequence of contamination by peptides, phenols, aromatic compounds or carbohydrates.

The integrity of the ribosomal RNA sub-units (23S, 16S and 5S for prokaryotes), the presence/absence of low weight RNA degradation products and the presence/absence of genomic DNA contamination are commonly visualized using agarose gel electrophoresis. Ideally, all expected ribosomal RNA sub-units should be observed, with no signs of RNA degradation products or presence of genomic DNA.

The guanidinium thiocyanate-phenol-chloroform extraction [8,9], commercially available as TRIzol (from Invitrogen) or TRI Reagent (from Molecular Research Center), is a frequently used method for cyanobacterial RNA extraction. This method, from this point referred to as Trizol, is usually associated with bead beating for physical disruption of the cells. In the present work we introduce the PGTX reagent, a reduced cost alternative to Trizol, and evaluate its use while exploring different extraction protocol variants.

## Results and discussion

### Extraction buffer (PGTX) formulation

The most important factor when planning the composition of the extraction buffer PGTX (detailed below) was to give the extraction solution the ability to quickly inhibit ribonuclease activity. Both phenol and guanidine salts are very efficient protein denaturants, therefore ideal for fast ribonuclease denaturation, and their combined use has been previously described [8,9]. We also added 8-hydroxyquinoline since it acts both as phenol stabilizer (preventing oxidation) and as RNase inhibitor [10].

The poor miscibility of phenol with water allows for easy phase separation at a later stage of the extraction procedure, but should be minimized at the beginning of the process. In order to avoid premature phase formation, glycerol was used to facilitate phenol solubility in the buffer. Later on, phase separation is achieved by adding BCP (bromochloropropane), as previously described [11].

After phase separation, protection of the extracted RNA is reduced, since the phenol and guanidine salt concentrations will be lower. In order to avoid degradation from this point on in the process, we used both sodium acetate and EDTA as chelators to prevent divalent cation catalyzed RNA degradation.

Triton X-100 is a non ionic detergent used for protein solubilisation, membrane permeabilisation and cell lysis. It has been previously demonstrated that its use, in combination with chloroform and heat, is a viable strategy for RNA extraction from both Gram-positive and Gram-negative bacteria [12]. This method has been further modified by replacing chloroform extraction with an acid phenol extraction [13]. For these reasons we included Triton X-100 in the PGTX extraction solution.

The PGTX solution has the following composition (for a final volume of 100 mL): phenol (39.6 g), glycerol (6.9 mL), 8-hydroxyquinoline (0.1 g), EDTA (0.58 g), sodium acetate (0.8 g), guanidine thiocyanate (9.5 g), guanidine hydrochloride (4.6 g) and Triton X-100 (2 mL), and the final pH around 4.2. At room temperature, the PGTX extraction mixture forms a monophasic solution.

### Evaluation of RNA extraction yield, purity and integrity

**Figure 1:**
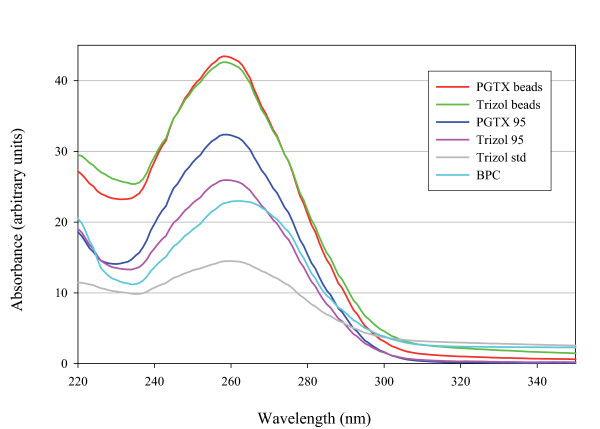
**Extraction contamination analysis**. Absorption spectra in the UV-region for purified RNA using 6 different extraction methods. For each method, 6 cyanobacteria aliquots were used for RNA extraction. From all obtained RNA samples, 1 μl was analysed using the NanoDrop ND-1000 UV/Vis spectrophotometer. The resulting lines shown are averaged from values obtained for each extraction method.

**Figure 2:**
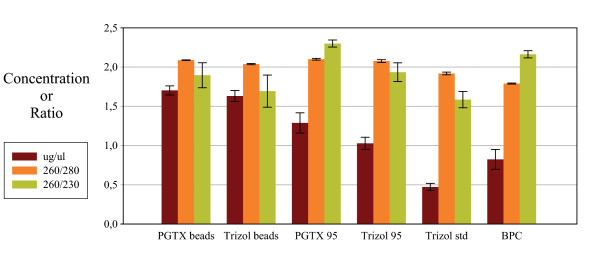
**Extracted RNA yield and purity**. Yield and absorption ratios for the different extraction methods were determined. For each method, 6 cyanobacteria aliquots were used for RNA extraction and 1 μl analysed using the NanoDrop ND-1000 UV/Vis spectrophotometer. The resulting bars shown are the average, and the standard deviation, from values obtained for each extraction method.

The strategies followed for RNA extraction are summarized in Table 1. During each experimental repetition, RNA was extracted from 6 cell aliquots (further information in Methods) for each of the methods investigated. The extracted RNA yield and purity was then determined by measuring absorbance in the 220 nm to 350 nm range (Figure 1). From the resulting spectra, the concentration of nucleic acids was estimated using the absorbance values at 260 nm, while the purity of each sample was determined by calculating the 260/280 and 260/230 ratios (Figure 2).

**Table 1:** Methods analysed

Method	Basic chemistry	Physical stress	Temperature stress	Yield (μg/ul)	260/280 ratio	260/230 ratio
PGTX beads	phenol, glycerol, guanidine, triton x	bead beating	N/A	1.70	2.1	1.9

Trizol beads	phenol, guanidine isothiocyanate (proprietary)	bead beating	N/A	1.63	2.0	1.7

PGTX 95	phenol, glycerol, guanidine, triton x	N/A	95°C	1.29	2.1	2.3

Trizol 95	phenol, guanidine isothiocyanate (proprietary)	N/A	95°C	1.03	2.1	1.9

Trizol std	phenol, guanidine isothiocyanate (proprietary)	N/A	N/A	0.47	1.9	1.6

BCP	phenol, chloroform	bead beating	N/A	0.82	1.8	2.2

Description, for each method analysed, of basic chemistry and cell lysis strategy followed. Bead beating and heat were used as described in Methods. (N/A – not applicable).

The highest yields were achieved using the "PGTX beads" and "Trizol beads" methods – averaging 1.70 μg/μl and 1.63 μg/μl respectively. For "PGTX 95" the yield was 1.29 μg/μl while for "Trizol 95" the calculated concentration was 1.03 μg/μl. The "Trizol standard" procedure yielded 0.47 μg/μl while the previously published method "BPC" resulted in a solution containing 0.82 μg/μl of nucleic acids. The different yields are probably correlated with the ability of each method to promote cell lysis. For instance, when considering the use of Trizol, we observed that combining it with bead beating is more effective than using heat or Trizol alone (see Table 1 and Figure 2). We also noted that, as opposed to bead beating, heating to 95°C does not result in an equal RNA yield for Trizol (1.03 μg/μl) and PGTX (1.29 μg/μl) – this difference is probably the result of composition differences that allow PGTX to promote more extensive cell lysis.

For the methods using PGTX, 260/280 ratios had a value around 2.0 – the expected value for a "pure" RNA sample. The same was observed for Trizol based methods, with a slightly lower ratio, close to 1.9, when following the procedure recommended by the manufacturer. For the "BPC" method the 260/280 ratio was 1.8, indicating putative DNA contamination.

Only for the "PGTX 95" and "BPC" methods were the 260/230 ratios around 2.2 – indicating a low levels of contaminantion with peptides, phenols, aromatic compounds or carbohydrates. For all other methods, these values ranged from 1.6 to 1.9. Noticeably, both the "Trizol beads" and "Trizol standard" presented a slight brownish coloration and an insoluble white precipitate (indicating some form of contamination in the samples). In Figure 1 this can be seen as a tail of absorption stretching out to 350 nm and beyond in the spectra from the three Trizol methods. Visible spectra up to 800 nm were also collected and only showed continuously decreasing absorbance (data not shown) and gave no further information about the origin of the coloured contamination.

**Figure 3:**
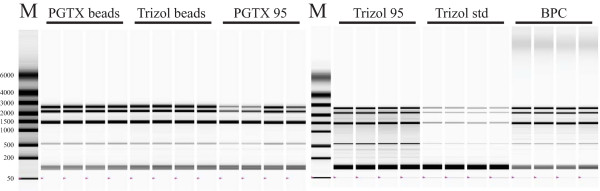
**RNA integrity checking**. Gel image generated by the automated electrophoresis system (Experion, Biorad). Lanes M – RNA molecular weight markers supplied with the Experion RNA StdSens analysis kit. Lanes PGTX beads – 1 μl of extracted RNA from distinct extractions using the PGTX solution combined with mechanical cell disruption with glass beads. Lanes Trizol beads – 1 μl of extracted RNA from distinct extractions using Trizol combined with mechanical cell disruption with glass beads. Lanes PGTX 95 – 1 μl of extracted RNA from distinct extractions using the PGTX solution combined with high temperature cell disruption. Lanes Trizol 95 – 1 μl of extracted RNA from distinct extractions using Trizol combined with high temperature cell disruption. Lanes Trizol std – 1 μl of extracted RNA from distinct extractions using Trizol according to general manufacturer's instructions. Lanes BPC – 1 μl of extracted RNA from distinct extractions using the BPC method. RNA integrity was kept for all extraction methods.

In order to verify integrity, four RNA samples for each of the extraction methods were analysed using an automated gel electrophoresis system (see Figure 3). For all methods the expected 23S, 16S and 5S bands are observed – while RNA integrity is kept for all extraction methods, the replicates of "BPC" presented high molecular weight smears. These were most probably genomic DNA, since they were absent after DNase digestion (data not shown).

### Detecting DNA contamination

**Figure 4:**
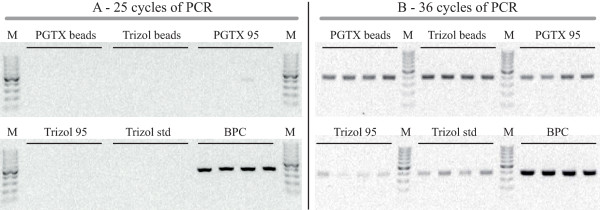
**DNA contamination detection**. Agarose gel separation of PCR products. For each of the six described methods, 20 ng of RNA from 4 different RNA extractions were used to perform PCR (using the "*ftsZ *sense" and "*ftsZ *antisense" primers). Resulting products were analysed after 25 (A) and 36 (B) cycles of PCR. Lanes M – double stranded DNA molecular weight markers (GeneRuler 100 bp DNA Ladder, Fermentas). To different extents, all extractions showed signs of genomic DNA contamination.

DNA contamination of RNA preparations is not necessarily detected by gel electrophoresis or similar methods. To test if a detectable amount of genomic DNA was present after simulated RT reactions (detailed in Methods), the diluted RNA samples were used as template for PCR using primers pairs for *ftsZ *(see Table 2). After 25 cycles of PCR (Figure 4A) only the "BPC" method resulted in detectable DNA contamination. But these findings were unexpected, since we expect some level of genomic DNA contamination for all extraction methods. In fact, after increasing the number of cycles from 25 to 36, more PCR products were detected (Figure 4B). Overall, the least DNA amplification was observed using the "Trizol standard" protocol, while using the "BPC" method results in the highest genomic DNA contamination.

**Table 2:** Primers used

Primer name	Sequence
*ftsZ *sense	CGAGATTGTCCCTGGTCGG

*ftsZ *antisense	TGGCTACTTCTGCTACGATTGGAG

*rbcL *tagged RT	CAACAGACGCACGACGCAGCAGACGAAACGGATATCTTCTAGAC

*rbcL *sense PCR	CGTTCCGCATGACACCCCAGCC

antisense TAG	CAACAGACGCACGACGCAGCAGAC

Sequence of primers used for this work. For genomic DNA detection *ftsZ *specific primers were used. During reverse transcriptase one tagged antisense oligonucleotide was used to prime cDNA synthesis. PCR amplification of the cDNA used one *rbcL *specific sense primer while the tag was used as an antisense primer.

### Performing RT-PCR

**Figure 5:**
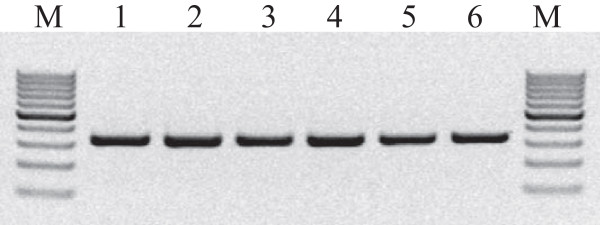
**RT-PCR suitable RNA extractions**. Agarose gel separation of RT-PCR products. For each of the six described methods, 1 μg of RNA was used as template for RT primed the "*rbcL *tagged RT" primer. Obtained cDNA was amplified using the "*rbcL *sense PCR" and "antisense TAG" primers. Lane 1 – template RNA obtained using the "PGTX beads" method. Lane 2 – template RNA obtained using the "Trizol beads" method. Lane 3 – template RNA obtained using the "PGTX 95" method. Lane 4 – template RNA obtained using the "Trizol 95" method. Lane 5 – template RNA obtained using the "Trizol std" method. Lane 6 – template RNA obtained using the "BPC" method. Lanes M – double stranded DNA molecular weight markers (GeneRuler 100 bp DNA Ladder, Fermentas). None of the extraction methods negatively impacted reverse transcriptase activity.

Reverse transcriptase is known to be inhibited by endonucleases, exonucleases and photosynthetic pigments [14,15], making RT-PCR a suitable test to determine if the extracted RNA is suitable for common downstream applications. Therefore, we performed RT-PCR using 1 μg of RNA from each extraction method as template to synthesize and amplify *rbcL *specific cDNA (Figure 5). To allow specific detection of mRNA, independent of genomic DNA contamination, we used tagged primers [16,17].

### Suggested protocol for RNA extraction

**Figure 6:**
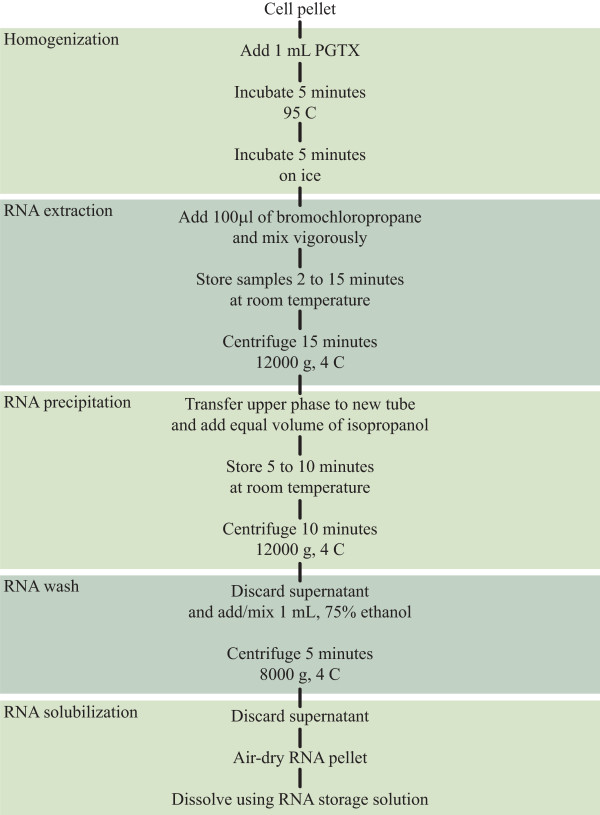
**Extraction protocol outline**. Schematic describing the recommend RNA extraction procedure.

In light of the previous results, for the best combination of yield and RNA extraction purity, we suggest the "PGTX 95" protocol outlined in Figure 6, a modification of the original Trizol protocol. Briefly, to a cyanobacterial cell pellet (not exceeding 100 mL) 1 mL of PGTX is added. After resuspending the cells, the screw-cap tubes are incubated at 95°C for 5 minutes. Immediately after, the samples are placed on ice, again for a period of 5 minutes. After addition of 100 μl of bromochloropropane and incubation at room temperature, the extraction mix is centrifuged in order to promote phase separation. The aqueous phase is then retrieved and mixed with an equal volume of isopropanol, incubated at room temperature and centrifuged to concentrate the precipitated RNA. The RNA pellet is washed using 75% ethanol, air dried and finally dissolved in RNA storage solution.

### Cost analysis for the different Methods

The cost per sample for the different procedures was estimated taking in consideration only the cost of each phenol based disruption solution, based on Sigma-Aldrich list prices for Sweden during February 2009. For the "BPC" method, the use of purchased buffer saturated phenol for RNA costs from €0.26 to €0.45 per extraction (depending on volume of reagent purchased). For the PGTX based methods the cost is €0.29 (assuming the prices for molecular biology grade reagents in the smallest available purchase volumes). For Trizol based extractions the cost per sample is €2.35.

## Conclusion

Cyanobacteria have an important amount of polysaccharides that interfere in cell lysis and nucleic acid purification [7,18], while being rich in nucleases and enzymatic reaction inhibitors [14,15,19-22]. With this work we show that variations of extraction protocol, even while maintaining basic chemistry, can have an impact on RNA yield and quality. While none of the extractions methods negatively impacted reverse transcriptase activity, we show that replacing bead beating with heating is feasible and maybe even preferable, as the level of contamination is lower and the method does not require the use of an expensive bead beater.

Potentially even more cost reducing is PGTX – this extraction mixture has equivalent potential to Trizol, while having only a fraction of the cost. We have also successfully used (data not shown) PGTX to extract RNA from several cyanobacteria (e.g. *Synechocystis *PCC 6803, *Anabaena *PCC 7120) and green algae (e.g. *Chlamydomonas reinhardtii*, *Chlamydomonas noctigama*).

## Methods

### Cyanobacteria growth conditions

Due to its secondary metabolite complexity and the presence of exopolysaccharides, *Nostoc punctiforme *was selected as target for RNA extraction. The hydrogen evolving NHM5 strain of *Nostoc punctiforme *ATCC 29133 [23] was grown in 500 mL cylinder-shaped flasks. Briefly, 400 mL of BG11 was inoculated, under sterile conditions, to an optical density of 0.2. The flasks were then connected to an air supply by a sterilised PE tube. The incoming air was pre-moistened and filtered with a PTFE membrane filter and the airflow set to 400 mL/minute. The cultures were illuminated with 58 W tri-phosphor fluorescent lamps (Aura, Karlskrona, Sweden) at a light intensity of 60 μmol m^-2^s^-1^. After 7 days of growth, circa 3.2 L of culture were centrifuged and pooled. The concentrated cells were aliquot into 2 mL screw-cap tubes and stored at -80°C until used for RNA extraction.

### RNA extraction using the "BPC" method

The extractions using the "BPC" method were performed as previously described [24], except that no 0.1 mm beads were used and the bead beating settings were not the same. All procedures were carried out at room temperature and centrifugations at 10000 g. The concentrated cell samples were centrifuged for 3 minutes and resuspended in 800 μl of water and 600 μl of buffer saturated phenol (pH 4.3). Addition of 0.5 g of 0.5 mm glass beads was followed by homogenization using a Bertin Precellys 24 (maximum speed, 20 seconds, twice). The sample tubes were then centrifuged for 10 minutes after which 750 μl of aqueous layer was transferred to a fresh tube, mixed with same volume of buffer saturated phenol (pH 4.3) and vortexed for 30 seconds. This was followed by centrifugation for 5 minutes and removal of 700 μl of aqueous supernatant that was then mixed with same volume of chloroform, in fresh tubes. After vortexing for 30 seconds, the samples were centrifuged for 5 minutes. At this point, 650 μl of aqueous layer were transferred to a fresh tube to which 65 μl of 3 M sodium acetate and 650 μl of isopropanol were added. The tubes were vortexed for 30 seconds and stored at -20°C for 10 minutes. After centrifugation for 5 minutes, the supernatant was removed and the pellet washed with 1 mL of 70% ethanol. One other centrifugation followed (5 minutes) and after removing the supernatant and air drying the RNA 50 μl of RNA storage solution (1 mM sodium citrate, pH 6.4, Ambion, Austin, USA) was added. The samples were stored at -80°C until analyzed.

### Trizol protocol based RNA extractions

For the "Trizol std" method the protocol provided by Molecular Research Center, was carried out without any modification [25]. The "Trizol beads", "Trizol 95", "PGTX beads" and "PGTX 95" extraction methods were based on this protocol, but the homogenization step was replaced either by bead beating (as described for the "BPC" method) or incubation at 95°C for 5 minutes. For the "PGTX" protocols, Trizol was simply replaced by the disruption mixture we propose in this paper.

### RNA quantity and quality assessment

RNA concentration and purity was measured using the NanoDrop ND-1000 UV/Vis spectrophotometer according to manufacturer's instructions (NanoDrop Technologies, USA). RNA integrity was verified using Biorad's automated electrophoresis system Experion (RNA StdSens analysis kit), according to manufacturer's instructions.

### PCR and RT-PCR

For DNA contamination detection, 1 μg of RNA was diluted in water to a total volume of 20 μl. Then, 1 μl of RNase A/T1 Mix (Fermentas) plus 1 μl of RNase H (Fermentas) were added and the samples incubated at 37°C for 30 minutes. To inactivate the RNases, 28 μl of TE buffer (pH 8.0) were added and the solution incubated at 70°C for 10 minutes. Using 1 μl of the described reverse transcriptase simulated reaction as template, PCR was carried out using specific primers for the division related gene *ftsZ *(see Table 2), using *Taq *DNA Polymerase (Fermentas), according to manufacturer's instructions.

To perform RT, 1 μg of RNA was used as template for RevertAid H Minus M-MuLV Reverse Transcriptase (Fermentas), according to manufacturer's instructions. In order to avoid the interference of genomic DNA contamination during the subsequent PCR step, a *rbcL *specific tagged primer [26,27] was used to primer the RT reaction (Table 2).

## Abbreviations

cDNA: complementary deoxyribonucleic acid; DNA: deoxyribonucleic acid; DNase: DNA ribonuclease; BCP: bromochloropropane; GSP: gene specific primer; mRNA: messenger ribonucleic acid; PCR: polymerase chain reaction; RNA: ribonucleic acid; RT-PCR: reverse transcription-polymerase chain reaction.

## Authors' contributions

FLP conceived the experimental setup and PGTX formulation, performed most experimental work and wrote most of the manuscript. AT performed spectrophotometric experiments, analyzed spectrophotometric data and actively contributed to the writing of the manuscript. WS was responsible for setting up culturing, harvesting and cell aliquoting, performed all BPC method extractions and participated in writing the manuscript. PL coordinated the project and contributed to the manuscript writing. All authors have read and approved the manuscript.

## References

[B1] Castenholz RW, Garrity GM (2001). Phylum BX. Cyanobacteria – Oxygenic Photosynthetic Bacteria. Bergey's Manual of Systematic Bacteriology.

[B2] Hansel A, Lindblad P (1998). Towards optimization of cyanobacteria as biotechnologically relevant producers of molecular hydrogen, a clean and renewable energy source. Applied Microbiology and Biotechnology.

[B3] Tamagnini P, Leitao E, Oliveira P, Ferreira D, Pinto F, Harris DJ, Heidorn T, Lindblad P (2007). Cyanobacterial hydrogenases: diversity, regulation and applications. FEMS microbiology reviews.

[B4] Lopes Pinto FA, Troshina O, Lindblad P (2002). A brief look at three decades of research on cyanobacterial hydrogen evolution. International Journal of Hydrogen Energy.

[B5] Singh S, Kate BN, Banerjee UC (2005). Bioactive compounds from cyanobacteria and microalgae: an overview. Critical reviews in biotechnology.

[B6] Dittmann E, Erhard M, Kaebernick M, Scheler C, Neilan BA, von Döhren H, Börner T (2001). Altered expression of two light-dependent genes in a microcystin-lacking mutant of *Microcystis aeruginosa *PCC 7806. Microbiology.

[B7] Tillett  D, Neilan BA (2000). Xanthogenate nucleic acid isolation from cultured and environmental cyanobacteria. Journal of Phycology.

[B8] Chomczynski P (1993). A reagent for the single-step simultaneous isolation of RNA, DNA and proteins from cell and tissue samples. BioTechniques.

[B9] Chomczynski P, Sacchi N (1987). Single-step method of RNA isolation by acid guanidinium thiocyanate-phenol-chloroform extraction. Analytical Biochemistry.

[B10] Emmett M, Petrack B (1988). Rapid isolation of total RNA from mammalian tissues. Analytical Biochemistry.

[B11] Chomczynski P, Mackey K (1995). Substitution of Chloroform by Bromochloropropane in the Single-Step Method of RNA Isolation. Analytical Biochemistry.

[B12] Sung K, Khan SA, Nawaz MS, Khan AA (2003). A simple and efficient Triton X-100 boiling and chloroform extraction method of RNA isolation from Gram-positive and Gram-negative bacteria. FEMS Microbiology Letters.

[B13] Phongsisay V, Perera VN, Fry BN (2007). Evaluation of eight RNA isolation Methods for transcriptional analysis in Campylobacter jejuni. Journal of Microbiological Methods.

[B14] Lau AF, Siedlecki J, Anleitner J, Patterson GM, Caplan FR, Moore RE (1993). Inhibition of reverse transcriptase activity by extracts of cultured blue-green algae (cyanophyta). Planta medica.

[B15] Cardellina JH, Munro MH, Fuller RW, Manfredi KP, McKee TC, Tischler M, Bokesch HR, Gustafson KR, Beutler JA, Boyd MR (1993). A chemical screening strategy for the dereplication and prioritization of HIV-inhibitory aqueous natural products extracts. Journal of natural products.

[B16] Shuldiner AR, Nirula A, Roth J (1990). RNA template-specific polymerase chain reaction (RS-PCR): a novel strategy to reduce dramatically false positives. Gene.

[B17] Smith RD, Ogden CW, Penny MA (2001). Exclusive amplification of cDNA template (EXACT) RT-PCR to avoid amplifying contaminating genomic pseudogenes. BioTechniques.

[B18] Wilkins TA, Smart LB, Krieg PA (1996). Isolation of RNA from plant tissue. A Laboratory Guide to RNA: Isolation, Analysis and Synthesis.

[B19] Cohen MF, Wallis JG, Campbell EL, Meeks JC (1994). Transposon mutagenesis of *Nostoc *sp. strain ATCC 29133, a filamentous cyanobacterium with multiple cellular differentiation alternatives. Microbiology.

[B20] Giovannoni SJ, DeLong EF, Schmidt TM, Pace NR (1990). Tangential flow filtration and preliminary phylogenetic analysis of marine picoplankton. Applied and environmental microbiology.

[B21] Golden SS, Brusslan J, Haselkorn R (1987). Genetic engineering of the cyanobacterial chromosome. Methods in enzymology.

[B22] Neilan BA (1995). Identification and Phylogenetic Analysis of Toxigenic Cyanobacteria by Multiplex Randomly Amplified Polymorphic DNA PCR. Applied and environmental microbiology.

[B23] Lindberg P, Schütz K, Happe T, Lindblad P (2002). A hydrogen-producing, hydrogenase-free mutant strain of *Nostoc punctiforme *ATCC 29133. International Journal of Hydrogen Energy.

[B24] Kim B-H, Oh H-M, Lee Y-K, Choi G-G, Ahn C-Y, Yoon B-D, Kim H-S (2006). Simple method for RNA preparation from cyanobacteria. Journal of Phycology.

[B25] TRI reagent – RNA/DNA/protein isolation reagent. http://www.mrcgene.com/tri.htm.

[B26] Pinto F, Svensson H, Lindblad P (2007). Webtag: a new web tool providing tags/anchors for RT-PCR experiments with prokaryotes. BMC Biotechnology.

[B27] Pinto F, Svensson H, Lindblad P (2006). Generation of non-genomic oligonucleotide tag sequences for RNA template-specific PCR. BMC Biotechnology.

